# Patient Comfort During Postop Period in Breast Cancer Surgeries: A Randomized Controlled Trial Comparing Opioid and Opioid-Free Anesthesia

**DOI:** 10.7759/cureus.33871

**Published:** 2023-01-17

**Authors:** Aravindhan Krishnasamy Yuvaraj, Balasubramaniam Gayathri, Natarajan Balasubramanian, Gunaseelan Mirunalini

**Affiliations:** 1 Anaesthesiology, SRM Medical College Hospital & Research Centre, Chennai, IND

**Keywords:** opioid free anesthesia, pain, emotions, quality of recovery, modified radical mastectomy, opioids

## Abstract

Background: Anesthetic management practices have advanced to opioid-free anesthesia (OFA) often replacing opioids in oncologic surgeries. The study was conducted to find the quality of recovery (QoR) of patients undergoing breast cancer surgeries receiving OFA.

Methods: A double-blinded, randomized controlled study was conducted with 60 patients randomized to group OFA and group OA (opioid anesthesia). Group OFA received one-time dosing of ketamine 0.3 mg/kg, lignocaine 1.5 mg/kg, and dexmedetomidine 1 mcg/kg. Group OA received fentanyl 2mcg/kg. Intraoperatively, Group OFA received dexmedetomidine 0.4 mcg/kg/h and the OA group received fentanyl 0.5 mcg/kg/h infusion. Bispectral index (BIS), hemodynamics, muscle relaxant administration, and sevoflurane concentration were noted. A modified QoR-40 score was used to assess the quality of recovery in the postoperative period.

Results: A higher QoR-40 score was found in the OA group (median 182, IQR 178-186) compared to the OFA group (median 180, IQR 178-184). Out of the five components, the patient's emotional state was better in the group OA (39.9±2.77) than in the OFA group (37.9±2.77). The patient's physical comfort was found to be better in the group OFA (52.52±3.23) compared to group OA (50.93±3.23). Physical independence, psychological support, and pain were comparable between the two groups. Patients in group OFA received less sevoflurane, a muscle relaxant, and showed a mild reduction in heart rate and mean arterial pressure (MAP) when compared to group OA. The time taken to reach a Modified Aldrete score of 9 was high in OFA (11.47±2.16) and (9.17±1.09) when compared to group OA. No significant differences were noted with the visual analog score (VAS) score, Ramsay sedation score, and modified post-anesthesia discharge scoring system (PADSS) score.

Conclusion: We conclude that the quality of recovery of patients receiving opioid-free methods of anesthesia was not inferior to OA in patients undergoing breast cancer surgeries.

## Introduction

Breast cancer is the second most common cause of cancer-related death among women worldwide [1]. Approximately 12% of women develop breast cancer. Specific treatment for the condition depends on the stage and type of tumor. Treatment for breast cancer includes surgery, radiation, and chemotherapy among which surgery plays a key role [2]. To date, intravenous opioids remain the primary agents used in pain management, and long-term postoperative pain control utilizing opioids has been approved by many countries [3].

Tolerance, development of opioid use disorder, death due to opioid misuse, and overdose among post-surgical patients have encouraged governments to develop strategies and call for further research to prevent the opioid crisis. Anesthetists worldwide have started considering opioid-free anesthesia (OFA) for day-to-day practice [4]. Although there is no concrete evidence, studies have shown that opioids may directly stimulate the proliferation and invasion of tumor cells and inhibit their apoptosis or indirectly affect cancer recurrence by immunosuppression. Genetic polymorphisms have been shown to affect opioid receptor function and modify the clinical effects of opioids. [5]. Pre-clinical data have also suggested that μ-opioid receptor inhibition can reverse the adverse effect of μ-opioid receptor signaling on cancer progression. In this randomized controlled trial (RCT) we compared two groups of patients, one receiving opioid fentanyl and the other a dexmedetomidine-ketamine combination with added lignocaine. Quality of recovery (QoR) in the first 24 of the postoperative period was evaluated using the quality of QoR 40 score [6,7]. We hypothesized that OFA will provide at least equal-if not better-postoperative comfort to the patients as opioid anesthesia (OA).

## Materials and methods

This prospective, double-blind, randomized, single-center study was conducted at a tertiary care hospital in South India. It was approved by our Local Institutional Ethics Committee (IEC 2410/15-04-2021). The trial was registered at Clinical Trials Registry (CTRI) (CTRI/2022/05/042925). The study was designed according to the Consolidated Standards of Reporting Trials (CONSORT) 2010 checklist.

Patients who underwent modified radical mastectomy between January 2022 and July 2022 were screened and those with an American Society of Anesthesiologists (ASA) physical status of III were included. The exclusion criteria were the following: allergy or contraindications to one of the study drugs, renal failure, hepatic failure, bleeding disorders, hyperthyroidism, atrioventricular block, heart rate (HR) <50 beats per min, valvular heart disease, left ventricular failure, ischaemic heart disease with ejection fracture <50%, unstable blood pressure, epilepsy, pregnant and lactating mothers, and psychiatric disturbance.

Patients satisfying the inclusion and exclusion criteria were informed about the anesthetic techniques and study methodology and written informed consent explaining both procedures was obtained. Patients were randomized to the OA and OFA using computer-generated random numbers. Group allocation was done using the sealed envelope technique. Patients were blinded to their allocated groups, and the observer in the operating room and nurses in the post-anesthesia care unit (PACU) were also blinded. The observer collected data (pre-, intra-, and post-operative) during the first 24 postoperative hours.

Demographic data including age, gender, ASA status, height, and weight of the patients were documented. Patient satisfaction was assessed pre- and postoperatively using the QoR-40 questionnaire. On the day of surgery, the patients were transferred to the premedication room, the intravenous line was secured and vital signs were recorded. Six hours of fasting for solids and two hours for clear liquids were ensured. To assess the physiological parameters, three lead electrocardiograms (ECG), non-invasive blood pressure (NIBP), pulse oximetry, and end-tidal carbon dioxide (ETCO2) monitors were attached. Furthermore, bispectral index monitoring (BIS) was utilized. The patients were pre-medicated with ondansetron 4 mg to prevent nausea and vomiting.

Patients were pre-oxygenated with 100% oxygen for three minutes. Those in the OA group received fentanyl 2 mcg/kg, while patients in the OFA group received dexmedetomidine 1 mcg/kg as an infusion over 10 minutes. In addition, patients in the OFA group received a bolus of ketamine 0.3 mg/kg and lidocaine 1.5 mg/kg. In both groups, induction was achieved with propofol 2 mg/kg and the trachea was intubated with an appropriate-sized tube using 0.1 mg/kg vecuronium as a muscle relaxant. Patients in both groups were given deep serratus anterior plane block (D-SAPB) using 0.25% ropivacaine 20 mL with 8 mg dexamethasone under ultrasound (USG) guidance. Anesthesia was maintained (Carestation 660, General Electric company, Chicago, Illinois, USA) with sevoflurane, the inhaled concentration of which was adapted to maintain hemodynamic stability and a BIS of 40-60. Upon surgical incision, acetaminophen (1000 mg) was administered intravenously to both groups. Patients in the OA group received fentanyl 0.5 mcg/kg/h infusion as part of the anesthetic, while those in the OFA group received dexmedetomidine 0.4 mcg/kg/h infusion. Muscle relaxation was achieved using 1 mg top-up doses of vecuronium whenever the train of four (TOF) count was one (STIMPOD NMS-40, XAVANT Technologies, Pretoria, South Africa). At the end of the surgical procedure, sevoflurane was discontinued, and once the TOF count was 4 and spontaneous breathing was ensured, the patient was reversed with 0.5 mg glycopyrrolate and 2.5 mg neostigmine. The patient was extubated when the TOF count reached 0.9%. Patients were transferred to the recovery room for further monitoring.

In the recovery room, each patient’s Aldrete score was assessed every five minutes until a score of 9 was reached; the time required to reach the score was also noted. Once the Aldrete score reached 9, the patient was shifted to PACU for continuous monitoring for 24 h. Postoperative pain relief in the OA group was ensured using fentanyl 0.5 mcg/kg/h, whereas the OFA group received dexmedetomidine 0.4 mcg/kg/h intravenous infusion. The time to achieve a modified PADSS (Post-anesthesia discharge score system) of 9 was assessed at intervals of six hours (6, 12, 18, and 24 h). The Ramsay sedation score was used to assess the sedation level. At the end of 24 hours, patients were asked to complete a questionnaire for assessing the quality of recovery. The QoR-40 consists of 40 questions evaluating emotional state, physical comfort, psychological support, physical independence, and pain [6]. Each question was scored from 1 to 5, and the maximum possible total score of 200. The lower the score, the higher the risk of complications (including extended hospital stay) and the lower the patient satisfaction. Preoperative baseline QoR-40 scores were recorded to identify cancer patients with a pre-existing altered psychological state, including anxiety.

To calculate the sample size, the mean difference in the pain component of QoR was obtained from the parent article by Honitor et al. [7]. The mean pain score of the OFA group was σ1=30.6, while that of the OA group was 28.46, with an α error of 5% and a power of 85%. Sample sizes of 58 with 29 were derived for each group. Data were entered in MS Excel Spreadsheet (2010) and were analyzed using the Statistical Product and Service Solutions (SPSS) (IBM SPSS Statistics for Windows, Version 21.0, Armonk, NY). Descriptive statistics including mean, standard deviation, median and interquartile range, were used to describe the data. Parametric continuous variables were compared using the independent sample t-test, while non-parametric continuous variables were compared using the Mann-Whitney U sample t-test. Significance was defined by P values less than 0.05 using a two-tailed test. Data analysis was performed using SPSS.

## Results

**Figure 1:**
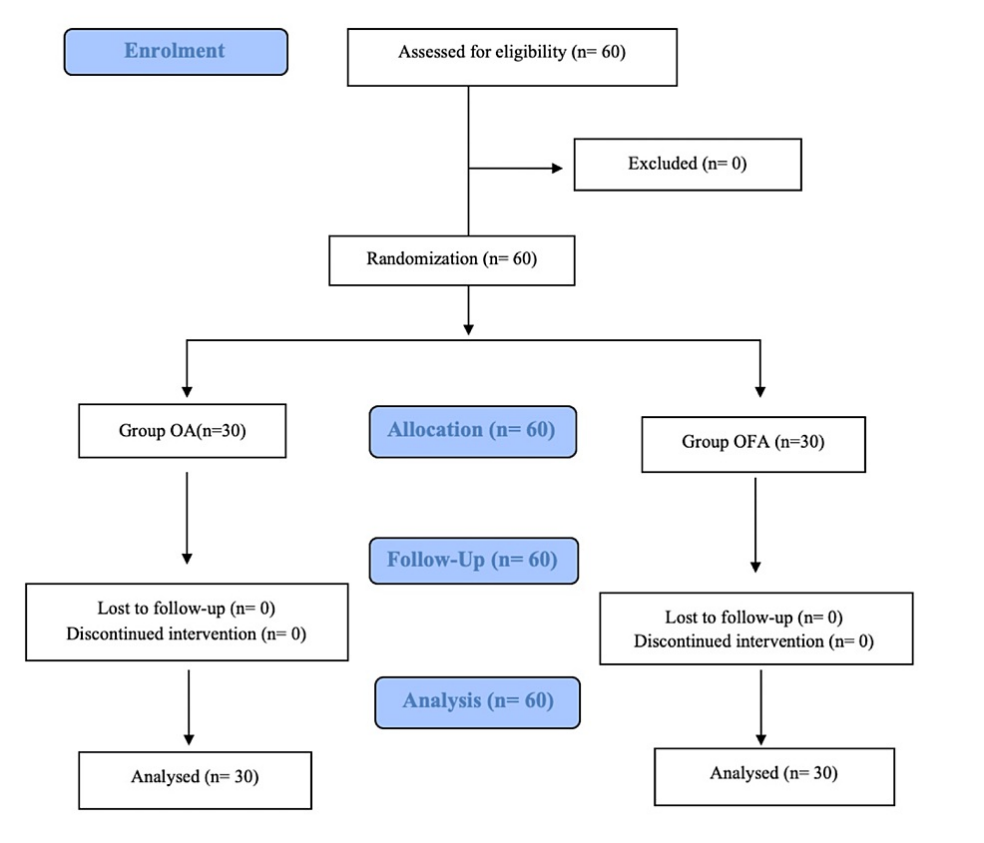
CONSORT flow diagram CONSORT: Consolidated Standards of Reporting Trials; OA: opioid anesthesia; OFA: opioid-free anesthesia

Sixty patients were enrolled, leading to a total number of 30 recruited patients in the OFA group, and 30 patients in the OA group (Figure 1).

Between the groups, no significant difference was noted in age, BMI, pre-operative QoR, or neo-adjuvant chemotherapy (Table 1).

**Table 1:** Demographic variables † - p value < 0.05 significant * - p value > 0.05 not significant BMI: body mass index; QoR: quality of recovery; OA: opioid anesthesia; OFA: opioid-free anesthesia

	Group				P value
	Group OFA		Group OA		
	Mean	STD	Mean	STD	
AGE	48.17	9.84	44.19	6.89	0.104*
BMI	23.23	2.34	24.07	3.10	0.245*
Neoadjuvant chemotherapy	21.33	2.2	20.33	2.1	0.0635*
Pre-operative QoR	181.78	6.5	183.44	6.3	0.6767*

The baseline HR was comparable between the groups. From the 15th minute significant fall in HR was noted in the OFA when compared to the OA group (73.60 ±7.13, 82.00 ±7.95; p-value <0.0001). The baseline mean arterial pressure (MAP) was comparable between the groups. From the 30th minute significant fall in MAP was noted in the non-opioid group when compared to the opioid group (86.00 ±7.23, 90.47 ±8.10; p-value <0.028). From the 15th-minute reading, the end-tidal sevoflurane concentration (ETS) titrated against MAP and to maintain BIS of 40-60 was found to be significantly lower in the OFA group compared to the OA group (1.25±0.35, 1.55±0.43 value <0.0001). The mean postoperative HR was found to be significantly less in the OFA group. There was a significant difference in the muscle relaxant consumption and time to reach a modified Aldrete score of 9 between the groups (Table 2).

**Table 2:** Distribution of muscle relaxant consumption and time taken to reach modified Aldrete score of 9 † - p value < 0.05 significant * - p value > 0.05 not significant OA: opioid anesthesia; OFA: opioid-free anesthesia

	Group				P value
	Group OFA (n=30)		Group OA (n=30)		
	Mean	Standard Deviation	Mean	Standard Deviation	
Muscle relaxant consumption	11.20	2.6	13.97	3.5	0.0005^†^
Modified Aldrete score of 9	11.47	2.16	9.17	1.07	<0.001^†^

The pre-operative QoR scores of patients in group OFA and OA were comparable. There was no significant difference in QoR scores within the group. A statistically significant difference in postoperative QoR-40 was observed between OFA (median 180, IQR 176-184) and OA groups (median 182, IQR 178-186; p-value <0.0001). On analyzing the individual components of QoR-40 scores, a statistically significant difference was observed in the emotional state with OFA (median 38, IQR 35-42) and OA (median 41, IQR 38-41). Similarly, we found a significant difference in physical comfort, OFA (median 52, IQR 50-55), and OA (median 51, IQR 48-54). OFA group patients had better scores in psychological support and physical independence, and the pain was better in the OFA group, although not statistically significant (Table 3).

**Table 3:** Post-operative QoR-40 score † - p value < 0.05 significant. * - p value > 0.05 not significant SD: standard deviation; IQR - interquartile range; QoR: quality of recovery

	Opioid-free group (n=30)	Opioid group (n=30)	P-Value
Emotional state			
Mean/Sd	37.79±2.77	39.49±2.2	0.007^†^
Median	38	41	
IQR	35-42	38-41	
Physical comfort			
Mean/Sd	52.52±3.04	50.93±3.23	0.04^†^
Median	52	51	
IQR	50-55	48-54	
Psychological support			
Mean±SD	31.97±1.74	32.39±2.27	0.388*
Median	32	33	
IQR	30-34	31-35	
Physical independence			
Mean±SD	23.06±1.84	23.03±1.81	0.94*
Median	23	23	
IQR	22-25	22-25	
Pain			
Mean±SD	30.00±2.7	28.3±4.7	0.87*
Median	30	28	
IQR	27.5-33	27-33	
Overall post-operative QoR-40 score			
Median	180	182	<0.07*
IQR	176-184	178-186	

No difference was noted in the visual analog score (VAS) score for pain or Ramsay sedation scores. Patients in both groups achieved a PADSS score of 9, in the sixth hour.

## Discussion

We studied patients undergoing modified radical mastectomy (MRM) for breast cancer and randomized to two groups. Group OFA received a single bolus of ketamine, lidocaine, and a continuous infusion of dexmedetomidine, while group OA received a continuous infusion of fentanyl. We found no difference in the QoR-40 scores between the groups during the first 24 h post-surgery.

The mean HR, MAP, and vecuronium doses were also significantly lower in the OFA group. We found that a combination of ketamine, lidocaine, and dexmedetomidine supplemented with deep serratus anterior plane (DSAPB) block can provide effective intraoperative analgesia. Studies have shown that opioid-based general anesthesia supplemented by nerve blocks provides good pain relief and maintains an adequate depth of anesthesia with a significantly lower end tidal sevoflurane (ETS) [8-10]. Cochrane review Pehora et al. cited that when used as an adjuvant to a peripheral nerve block, dexamethasone may prolong the duration and reduce pain intensity [11].

A study by Di Benedetto et al. in patients for breast cancer surgery comparing opioid and non-opioid groups found better pain relief in patients receiving OFA [12]. In our study patients in both groups had pain comparable scores, indicating no difference in pain intensity. In their comprehensive review of opioid-induced hyperalgesia, Lee M et al. comment that opioid-based anesthesia is linked to the development of acute pain tolerance. Acute pain tolerance can lead to an increase in the intensity of post-surgical pain [13].

Muller et al. conducted a study evaluating the effects of opioid-free versus opioid-general anesthesia using QoR-40 in patients undergoing laparoscopic bariatric surgery. They concluded that postoperative nausea, vomiting, and hypertension were higher in the opioid group. OFA group had a higher QoR-40 indicating that the QoR was better in the opioid-free group [14]. We observed that QoR-40 in both OA and OFA groups was comparable. In their study, Di Benedetto et al. comparing opioid and non-opioid anesthesia found that opioid-free treatment did not worsen patients’ overall satisfaction or comfort when compared with an opioid-inclusive approach [12]. A study by Kim et al. on intraoperative dexmedetomidine infusion on emergence agitation and quality of recovery after nasal surgery showed infusion of 0.4 mcg/kg dexmedetomidine improved the QoR-40 score measured 24 h post-surgery [15].

We found a significant difference in the patients’ emotional states between groups. Those in the OFA group had a median score of 38/45 (IQR 35-42), while those in the OA had a higher score of 41/45 (IQR 38-41). The parameters used to assess emotional states included various feelings from general well-being to anxiety, anger, and depression (feeling comfortable, having a general feeling of well-being, feeling in control, difficulty falling asleep and having bad dreams, feelings of anxiety, anger, or depression). We found that patients in the opioid group felt better emotionally than those in the opioid-free group. Studies have found an increase in euphoria or pleasure upon administration of μ receptor agonists. Nummenmaa et al. studied the effects of opioids on various emotions and concluded that opioid agonists strengthen approach-oriented emotions (anger, pleasure, socialization, and bonding) and weaken withdrawal-oriented emotions (fear and sadness) [16]. It is possible that this mood-altering property resulted in the patients receiving opioids feeling emotionally better than those in the OFA group [16].

Patients in the OFA group had better scores in the physical comfort component which included nausea and vomiting. Jan et al., in their study on 50 patients undergoing laparoscopic bariatric surgery, found that the QoR-40 score was lower in the opioid group owing to the increased incidence of postoperative nausea and vomiting (PONV). Di Benedetto et al. and Hontoir et al. also found the incidence of PONV was reduced in patients who received OFA [7,12]. Another component of physical comfort in the QoR-40 score is sleep. Donghee Kang et al. conducted a study on dexmedetomidine sedation and found that there was a correlation between the change in HR during natural sleep and dexmedetomidine sedation, indicating that dexmedetomidine sleep simulates natural sleep [17]. A natural sleep achieved with dexmedetomidine may be responsible for better physical comfort in the OFA group.

Limitation

Our study included a small group with no blinding. The used fixed dial settings for sevoflurane and the inhalational agent were not blinded per Minimum Alveolar Concentration (MAC) age. A BIS of 40-60 during the intra-operative period and the actual values were not captured for analysis. This is a limitation as a BIS of 40-60 is a wide range having implications on hemodynamics, sedation, and recovery. Possible observer bias was avoided by blinding the investigator and analyzing the QoR-40 score. Twenty-four hours long time is not enough to speculate reasons for improvement or worsening of quality of life. Repeating the questionnaire at the time of discharge could have added valuable insights. We also did not include participants at risk for whom the clinical trial will be unsafe.

## Conclusions

The nonopioid nerve block technique is adequate and safe for MRM. Except for the emotional and physical comfort, the QoR of patients not receiving opioids is good. OFA provides good analgesia preventing the side effects associated with opioid administration such as nausea and vomiting. Larger studies are needed to assess the long-term impact on chronic pain and tumor recurrence by nonopioid techniques.
